# Therapist Driven Rehabilitation Protocol for Patients with Chronic Heart and Lung Diseases: A Real-Life Study

**DOI:** 10.3390/ijerph17031016

**Published:** 2020-02-05

**Authors:** Carla Simonelli, Michele Vitacca, Nicolino Ambrosino, Simonetta Scalvini, Francesca Rivadossi, Manuela Saleri, Aubin G Fokom, Ilaria Speltoni, Riccardo Ghirardi, Mara Paneroni

**Affiliations:** 1 Istituti Clinici Scientifici Maugeri IRCCS, Cardiac Rehabilitation of the Institute of Lumezzane, 25065 Lumezzane (BS), Italy; carla.simonelli@icsmaugeri.it (C.S.); simonetta.scalvini@icsmaugeri.it (S.S.); francesca.rivadossi@icsmaugeri.it (F.R.); ilaria.speltoni@icsmaugeri.it (I.S.); 2 Istituti Clinici Scientifici Maugeri IRCCS, Respiratory Rehabilitation of the Institute of Lumezzane, 25065 Lumezzane (BS), Italy; michele.vitacca@icsmaugeri.it (M.V.); manuela.saleri@icsmaugeri.it (M.S.); georges.fokom@icsmaugeri.it (A.G.F.); riccardo.ghirardi@icsmaugeri.it (R.G.); 3 Istituti Clinici Scientifici Maugeri IRCCS, Respiratory Rehabilitation of the Institute of Montescano, 27040 Montescano (PV), Italy; nico.ambrosino@gmail.com

**Keywords:** cardiac rehabilitation, pulmonary rehabilitation, exercise, training, algorithm, physiotherapist protocol, driven protocols

## Abstract

Therapist driven protocols may help to tailor rehabilitation programs to individual patients. We aimed to test the feasibility, safety, and clinical usefulness of a therapist driven protocol for rehabilitation including exercise training of patients with heart or lung diseases. An internal audit elaborated the Cardio-Respiratory Exercise Maugeri Algorithm (CREMA) based on: (a) standardized baseline assessments, (b) decision-making pathways, and (c) frequency/intensity/time/type (FITT) of prescription for each exercise. Outpatients (*n* = 620) with chronic heart disease (CHD), recent myocardial revascularization (REVASC), chronic airway (Obstructive), and restrictive lung (Restrictive) diseases underwent exercise training according to CREMA during 4 years. Peripheral muscle strengthening was the most prescribed exercise (83.6%), while arm endurance training was the least frequently (0.75%). Exercise prescription varied widely among the disease groups (interval training 19–47%, balance 35–49%, lower limb muscle training 6–15%). After training, REVASC patients were the best improvers in the 6 min walking distance (+48.7 (56.1) m), maximal inspiratory pressure (+9.6 (15.4) cmH_2_O), and daily steps (+1087.2 (3297.1) n/day). Quadriceps and biceps strength, maximal expiratory pressure, and balance improved in all groups, without significant differences. Minor side effects were observed in 11.2% of the patients. The CREMA therapist driven protocol was feasible, safe, and useful for prescribing tailored training programs. Exercise prescriptions and training response differed among diseases.

## 1. Introduction

Rehabilitation including exercise training is an essential component of the comprehensive management of patients with lung and/or heart diseases [1,2,3,4,5]. There is a large body of evidence showing the benefits of rehabilitation including exercise training on symptom improvement, exercise tolerance, and health related quality of life [1,2,6]. However, due to the high prevalence of these diseases, the majority of potential candidates have no access to supervised programs [7,8]. In the attempt to save resources, allowing a larger delivery to patients, such programs have been proposed for patients with heart or lung disease, as they share similar symptoms and comorbidities [9,10]. A recent position paper stated that common training programs administered to patients with heart or lung disease are feasible, safe, and cost-effective for healthcare commissioners, thus improving the access to rehabilitation [11]. There is a lack of access to supervised programs, and the risk of over-standardization for patients with different diseases in such programs could be a problem. The implementation of a therapist driven protocol could reduce this risk. 

A driven protocol is defined as a consensus of medical knowledge and opinions based on an operative flow chart according to objective measurable variables and rigid items to reduce operator anarchy in prescription [12]. Driven protocols may help to tailor these programs to the individual patients. 

In cardiac field, therapist driven protocols, including tools to evaluate the main targets of exercise-based cardiac rehabilitation, have recently been proposed and decision support systems assisting healthcare professionals to prescribe training programs has been developed [13,14]. In these decision support systems [13,14], the authors have proposed only training as the main outcome measure referring to specific disease. Potential limitations of this approach have generated our hypothesis: to propose a new therapist driven protocol for patients with chronic heart and lung diseases including multiple outcomes. 

The aims of the study were: (1) to describe the audit process in order to develop a tailored therapist driven protocol for rehabilitation programs, including exercise training, using a battery of evaluation tools common to patients with heart or lung disease; (2) to test its feasibility and safety; and (3) to evaluate the clinical results.

## 2. Materials and Methods

This was a pragmatic study, conducted in the Cardio-Pulmonary Physiotherapy Service of the Cardiac and Pulmonary Rehabilitation Departments of the Istituti Clinici Scientifici (ICS) Maugeri IRCCS, Lumezzane (Brescia), Italy, between January 2015 and December 2018. The Ethics Committee approved the study (CE 2287,14 May 2019). All patients signed an informed consent to the scientific use of their clinical data. 

### 2.1. Development of the Protocol

An audit was created (from September to December 2014) in order to review decision-making pathways for training and rehabilitation programs. The audit was composed by a multidisciplinary expert panel (one senior and three junior physiotherapists, two cardiologists, and one pulmonologist). Experts met twice for an audit and were asked to define: (1) the required baseline assessments; (2) the frequency, intensity, time, and type (FITT) of exercise training to be included in the program; and (3) the decision-making pathways to define how the baseline assessment should influence the FITT prescription of each component of the program [15]. Prior to the meeting, a systematic review of the literature was performed, using the keywords: “pulmonary rehabilitation; cardiac rehabilitation, exercise training, inspiratory muscle training, strength training, balance training, physical activity, COPD, idiopathic pulmonary fibrosis, coronary artery disease, chronic heart failure”. Experts discussed their real-life experience. During the audit, a driven protocol and operative flow charts were summarized and proposed as a new operational bundle hypothesis based on the literature and consensus. The protocol was a tool for prescription of tailored rehabilitation programs according to the guidelines. 

**Figure 1:**
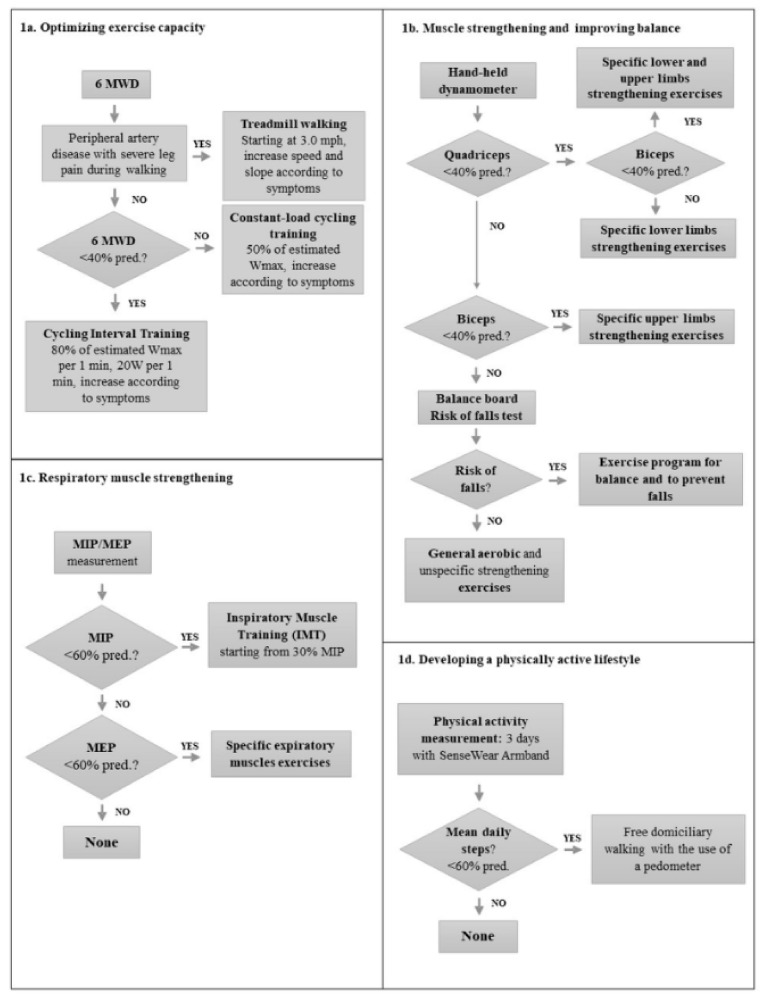
The algorithm developed in the audit process and used as a guide to train subjects with heart and lung diseases. 6MWD: 6-min Walk Distance; MEP: Maximal Expiratory Pressure; MIP: Maximal Inspiratory Pressure.

The experts scored the final protocol and flow charts using a Delphi-like procedure [16]. Each items and pathways were deemed approved if at least 75% of the group rated the proposal with a rating higher than 7 (0 = totally disagreed; 10 = totally in agreement). Finally, the audit approved the Cardio-Respiratory Exercise Maugeri Algorithm (CREMA) to be used as a guide for prescription of a therapist driven protocol in patients with heart and lung diseases (Figure 1).

A dedicated computerized chart was created. In detail:

a. The proposed measurements were:

1. 6 min walking distance (6MWD), according to the American Thoracic Society/European Respiratory Society (ATS/ERS) guidelines [17], using the predicted values by Chetta et al. [18]. The minimum clinically important difference (MCID) for 6MWD is 30.5 m [19]
2. Maximal voluntary contraction (MVC) of biceps and quadriceps muscles through hand-held dynamometry using the predicted values by Andrews et al. [20]; the MCID for quadriceps and biceps strength is 0.76 kg [21]. Muscle weakness was defined as MVC < 60% of the predicted values
3. Maximal inspiratory (MIP) and expiratory pressures (MEP), according to the ATS/ERS guidelines [22]; the predicted values were those by Black and Hyatt [23]
4. Stability index and risk of fall index, assessed by means of a balance board, with the predicted values of Cho et al. [24]
5. Domiciliary physical activity assessing daily steps [25]. The MCID is an increase of 1100 steps/day [26,27].

b. The proposed programs were:

1. Aerobic training (moderate–high intensity continuous or interval cycle training, treadmill walking, arm ergometer) [1,6]
2. Calisthenics [1,2,6,7,8]
3. Lower and/or upper limb selective muscle strengthening [1,6]
4. Balance training including core stability training, standing on unstable surfaces, balance boards, walking on tips and heels [28]
5. Inspiratory muscle training [29,30,31,32]
6. Domiciliary walking with pedometer [33,34].

For further information on CREMA development see Supplementary Materials.

### 2.2. Application of the CREMA

Our physiotherapists prospectively (from January 2015 to December 2018) used the CREMA as a guide to set an individually tailored supervised exercise program consisting of 20 sessions, with 2 to 4 per week, lasting 2 h each. The first two sessions were dedicated to baseline assessments; the results were used to set the program according to the CREMA, personalizing the components, and FITT prescribed for each individual. Initial exercise intensity was set by CREMA and progression of intensity of each component of prescribed exercises in subsequent sessions was based on symptoms of dyspnea and fatigue as assessed by the Borg scale [35] according to Maltais et al. [36]. Intensity of exercise was increased when Borg scale for fatigue and dyspnea was lower of 5 at the end of each session. All patients in each diagnosis group underwent the same assessments, which were repeated at the end of training. The number and percentage of patients prescribed each single component was collected. All training sessions took place in the gym room under physiotherapist supervision. In addition, all patients attended an educational session once a week to learn about chronic disease management, diet, drugs, and healthy lifestyle. Medications were optimized at the beginning of the program. A multidisciplinary team composed of physiotherapists, physicians, a psychologist, nutritionist, and occupational therapist took care of diet, airway clearance, psychological or social issues, and other aspects.

### 2.3. Feasibility and Safety 

Feasibility was investigated in terms of: (i) the type and number of prescriptions administered; (ii) the number of cases in which the physiotherapist had to change the initial prescription. Safety was investigated in terms of major and minor side effects. Major side effects were defined as events threatening the health of the patient requiring interruption of the program for specialist consultation and/or medical intervention. Minor side effects were defined as meaningful events of lower clinical severity inducing the stop of training for a maximum of 1–2 sessions, possibly but not necessarily requiring an adjustment of drug therapy [37].

For details on safety, feasibility and sensibility of CREMA see Supplementary Materials. 

### 2.4. Data Management and Statistical Analysis

Data were collected from the Automated Integrated Health Care Record (AIHCR). Results are expressed as mean (standard deviation) for continuous data and as percentage for categorical and binary ones. All subjects were divided into 4 groups according to the main discharge diagnosis requiring the highest health resources consumption under the ICD9 classification:

- Obstructive: Patients with chronic obstructive pulmonary disease (COPD) with or without chronic respiratory failure, or asthma according to the ICD9 classification. COPD was defined according to the Global Initiative for Chronic Obstructive Lung Disease (GOLD) criteria [38], and asthma according to the Global Initiative for Asthma (GINA) criteria [39]. In patients with lung diseases, dynamic and static lung volumes were assessed according to the American Thoracic Society (ATS) guidelines [40] by means of a body plethysmograph or a water-sealed spirometer. The predicted values were those of Quanjer et al. [41].
- Restrictive: Patients with interstitial lung diseases, obesity hypoventilation syndrome, kyphoscoliosis, or mixed diagnoses with forced vital capacity (FVC) <80% of predicted and with Forced Expiratory Volume at 1 s (FEV_1_) /FVC > 70%.
- Chronic heart disease (CHD): Patients with chronic heart failure (CHF), according to the 2016 European Society of Cardiology guidelines [42], and coronary artery disease (CAD), according to the criteria of the European Society of Cardiology [43].
- REVASC: Patients that have undergone a coronary artery by-pass graft (CABG) or percutaneous transluminal coronary angioplasty (PTCA) in the previous 3 months.

Cardiac and respiratory comorbidities were also recorded from the main secondary discharge diagnoses according to the ICD9 classification. 

Patients with primary diagnosis not included in the above groups (e.g., oncological, neurological, neuromuscular, locomotor diseases) or with the aforementioned diagnoses with locomotor limitations or contraindications to exercise were excluded.

Baseline data were compared among the different diagnoses, and the percentage of patients showing a severe impairment was calculated as follows. For 6MWD, quadriceps and biceps MVC, MIP, MEP, and balance, severe impairment was defined as a value <60% of the predicted. Reduced physical activity was defined as <3300 steps/day. The percentage of improvers in each measure was compared by means of X^2^ test.

Differences between baseline characteristics and outcome measures for each group were evaluated by one-way analysis of variance (ANOVA) for continuous variables, and by X^2^ evaluation for categorical ones. A pairwise post-hoc analysis was performed if the primary evaluation was significant using Bonferroni correction. A *p*-value lower than 0.05 was considered significant.

## 3. Results

**Figure 2:**
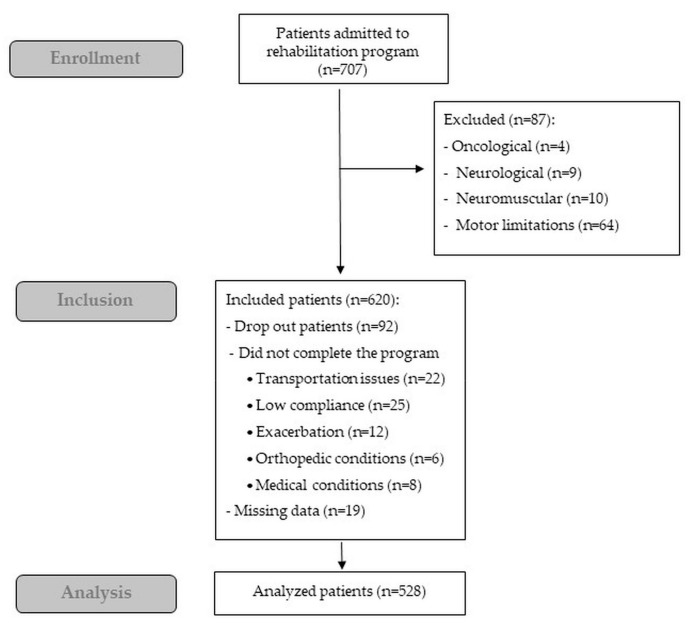
Flow diagram of the study.

Figure 2 shows the flow diagram of the study. Of the 707 outpatients (384 with lung and 323 with heart diseases) admitted during the study period, 87 patients were excluded. Data of 92 of the 620 included patients were not analyzed for different reasons. Therefore, data of 528 patients were analyzed.

Table 1 shows the characteristics of the patient sample. Cardiac comorbidities were observed in 19.7% of respiratory patients while 11.0% of cardiac patients suffered from respiratory comorbidities. Patients with heart diseases had higher 6MWD, quadriceps, biceps, and expiratory muscle strength than those with lung diseases. The Restrictive group was the most severely impaired, such that more than half of those patients had a severe reduction in the 6MWD, and one-fifth had a reduced balance index; the difference was significant compared to the two cardiac groups (CHD and REVASC; Table 2).

### Feasibility and Safety

The mean time needed to complete the baseline assessment, insert data into the computerized chart, and obtain the training prescription was 40.3 (8.5) min per patient. Patients performed 20.2 (5.4) training sessions, and 538 patients (86.8%) concluded the program without any need to change the exercise prescription. In 56 patients (9.0%; Obstructive 8.7%, Restrictive, 18.2%, CHD 8.4% and REVASC 4.4% *p* = 0.067), the intensity of exercise was reduced during the program, while in 38 (6.1%) some components of the training program (inspiratory muscle training or domiciliary walking) were not administered due to low compliance without any significant difference among groups. 

The number and type of components of the exercise program administered to each group are reported in Table 3. No major side effect was observed. Minor side effects occurred in 69 patients (11.1%) with 38 episodes of oxygen desaturation, 4 episodes of frequent ectopic ventricular beats, and 17 cases of abnormal arterial blood pressure (lower than 80/50 or higher than 160/110 mmHg) during exercise. None of these episodes resulted in interruption of the program. There was no significant difference in the number of patients experiencing side effects among groups.

Table 4 shows the post training changes in outcome measures, as delta score (absolute values) and percentage of baseline. The changes differed among diagnosis groups, with REVASC improving 6MWD and MIP more than the Obstructive group. Only the REVASC group increased the mean daily steps, while the respiratory groups actually worsened.

Overall, 69.9%, 63.8%, and 61.2% of patients improved more than the MCID for quadriceps MVC, biceps MVC, and daily steps, respectively. There was no significant difference among groups in the percentage of improvers in quadriceps or biceps MVC, or daily steps. REVASC group showed the highest percentage of improvers in 6MWD (50.6%) when compared to other groups (Obstructive 34.1%, Restrictive 37.5%, and CHD 37.8%). 

After the program, the male patients improved their MEP (by 5.3 (1.5%) vs 2.3 (12%), *p* = 0.037) and biceps strength (10.3 (19.7%) vs 6.0 (17.5%), *p* = 0.023) more than females. No other differences between males and females were found in post program changes. 

## 4. Discussion

This study shows that the therapist driven protocol, based on a common battery of evaluations, including exercise training, tailored to individual patients with heart or lung diseases is feasible and safe. A recent survey study carried out in the Netherlands showed that there is poor implementation of cardiac rehabilitation guidelines in daily practice [12], despite the evidence that large-scale implementation of a clinical protocol for cardiac rehabilitation can lead to a substantial increase in guideline adherence [44]. 

In previous decision support systems [13,14] the authors proposed only training, as main outcome measure referring to specific diseases. A therapist driven protocol like that produced by CREMA might allow better adherence to guidelines, and individually tailored programs with multiple evaluations and outcomes. The computerized decision support system has also increased the information available on the response to different prescriptions, providing feedback to improve the quality of the treatment [14]. 

Using the CREMA led to overall differences in the type of programs and exercises indicated for patients, with a substantial portion of exercise devoted to interval and balance training (Table 3). With the CREMA it was also possible to deliver different prescriptions among the different diagnoses. For example, inspiratory muscle training was prescribed only in 18.4% of the Obstructive group, i.e., those with MIP <60% predicted. This is in accordance with recent findings of clinical trials in patients with COPD [45]. 

To our knowledge, no previous studies have the changes in functional and physical status after rehabilitation across different diseases. We found that REVASC patients had a more preserved functional capacity, which improved in the majority of patients, with the highest percentage of improvers in 6MWD. An explanation may be that the REVASC patients did not suffer from any chronic condition when they entered the training program after an acute event. 

The only between groups difference in post program improvement in outcome measures was observed in inspiratory muscle strength which was significantly higher in CHD as compared to Restrictive patients. This is in line with Evans et al. [10], who did not find any difference in the response to training between chronic airway obstruction and CHF, and suggests that chronic diseases, irrespective of the diagnosis, may share some impairments of the systems involved in exercise performance, such as peripheral muscle dysfunction, respiratory muscle overload, cardiopulmonary limitation, and comorbidities. Otherwise Restrictive patients were the group with a minor response to rehabilitative intervention presenting the lowest mean baseline values in respiratory and peripheral muscles (Table 1), with the highest prevalence of impairment (<60% of predicted, Table 2), indicating a generalized and severe muscle dysfunction. 

In our study, REVASC patients improved daily steps more than those with pulmonary diseases. A huge variability has been reported in changes in physical activity after pulmonary rehabilitation [33,34]. Considering the overall results, given the high variability of the improvement in daily steps, both in pulmonary and cardiac diseases, we think that a better behavioral approach aimed at improving lifestyles and reducing sedentary behavior in chronic patients should be implemented and added to the training program. 

Only 34% of our patients with COPD were responders to training improving the MCID of 6MWD. At the best of our knowledge, randomized controlled trials of exercise training in patients with COPD report about one-third of non-responders, and many papers have been dedicated (mostly unsuccessful) to find any predictive factor of success. Our lower percentage of improvers as compared to previous literature may be explained on the basis of the more restrictive criteria of improvement we used. This is one of the reasons leading to search for multifactorial outcomes after rehabilitation and tailored programs [46]. Previous literature has shown that the percentage of non-responders to training programs ranged from 14% to 23% in patients with CAD or CHF [47,48,49].

Cardiac [1] and respiratory rehabilitation programs [2] are safe, with a low incidence of major events but, to our knowledge, no studies have assessed the safety of a common training program for cardiac and respiratory patients combined. Our program was safe and the minor side effects observed in a few patients are in line with similar programs proposed in the aforementioned patients. 

### 4.1. Limitations of the Study

As a retrospective and real-life study, we defined groups according to the administrative ICD9 classification. The reported comorbidity occurrence in our study must not be considered as a real prevalence as our patients did not undergo any specific diagnostic test. Furthermore, we did not assess the post exercise impact of the diseases or health related quality of life. 

### 4.2. Practical Implication

The CREMA protocol based on present guidelines is useful for the assessment and prescription of training, as part of the multidisciplinary approach for personalized management of cardiac and pulmonary diseases, based on thorough assessment of treatable traits [50]. Protocols driving the training prescription may be included in the routine practice to administer exercise, ensuring adherence to the guidelines, and customizing the treatment to the individual patient. 

## 5. Conclusions

Based on a common set of evaluations, our therapist driven protocol to tailor rehabilitation programs in patients with heart or lung diseases was feasible and safe. There were differences according to diagnoses, both in terms of functional capacity at the beginning of the program and the results obtained, with a better outcome in patients with recent myocardial revascularization than in those with chronic cardiac or respiratory diseases.

Future studies are needed to demonstrate whether this instrument will play an important role in achieving the aims of health care including better outcomes, lower cost of care, and improved experience for patients and staff. 

## Figures and Tables

**Table 1:** Demographic, anthropometric, physiological, and clinical characteristics of patients. Data are expressed as number (%) or mean (SD).

	All	Obstructive (O)	Restrictive (R)	CHD	REVASC (RE)	*p*-Value
*N* (%)	620	320 (51.6)	55 (8.9)	154 (24.8)	91 (14.9)	
		COPD = 205 COPD + CRF = 67 Asthma = 48	ILD = 10 OHS = 6 Other RS = 39	CHF = 36 CAD = 118	PTCA = 59 CABG = 32	
Age, years	67.1 (10.5)	68.3 (9.0)	65.1 (14.2)	67.6 (9.9)	63.0 (12.7)	<0.001 (RE vs. O <0.001; RE vs. CHD = 0.006)
Male, *n* (%)	430 (69.3)	224 (70)	26 (47.3)	109 (70.9)	71 (78.0)	0.001 (R vs. O = 0.006; RE vs. R <0.001; CHD vs. R = 0.012)
BMI, Kg/m^2^	27.9 (10.4)	27.2 (13.5)	27.4 (7.6)	29.4 (5.3)	28.0 (4.3)	0.160
FEV_1_, %pred		62.5 (23.1)	69.9 (32.9)			
FVC, %pred		88.6 (20.5)	72.6 (31.2)			
FEV_1_/FVC, %		71.2 (20.3)	92.1 (20.3)			
LVEF, %				55.6 (11.3)	57.8 (7.3)	
6MWD, meters	442.5 (108.6)	434.2 (106.0)	391.4 (110.8)	447.3 (109.1)	492.3 (97.2)	<0.001 (R vs. O = 0.04; RE vs. O <0.001; CHD vs. R = 0.006; RE vs. R <0.001; RE vs. CHD = 0.006)
6MWD, %pred	64.4 (14.7)	63.2 (14.8)	57.8 (15.8)	65.8 (14.8)	69.9 (11.5)	<0.001 (RE vs. O <0.001; CHD vs. R = 0.006; RE vs. R <0.001)
Quadriceps MVC, Kg	30.8 (11.3)	29.8 (10.4)	25.7 (8.9)	32.0 (12.4)	35.5 (12.1)	<0.001 (RE vs. O < 0.001; CHD vs. R <0.001; RE vs. R <0.001)
Quadriceps MVC, %pred	80.3 (26.1)	79.7 (25.6)	72.4 (27.6)	81.0 (26.1)	85.63 (26.6)	0.030 (RE vs. R = 0.02)
Biceps MVC, Kg	21.3 (7.5)	20.8 (7.3)	19.0 (7.2)	22.1 (7.8)	23.1 (7.2)	0.003 (CHD vs. R = 0.04; RE vs. R = 0.006)
Biceps MVC, %pred	92.4 (23.1)	92.56 (24.4)	90.37 (23.49)	92.71 (22.15)	92.54 (20.26)	0.955
MIP, cmH_2_O	74.1 (24.6)	74.2 (22.6)	64.0 (24.1)	74.6 (27.8)	78.7 (24.4)	0.006 (R vs. O = 0.02; CHD vs. R = 0.04; RE vs. R < 0.001)
MIP, % pred	78.3 (24.0)	79.4 (23.6)	73.7 (26.8)	77.5 (24.9)	78.34 (22.1)	0.426
MEP, cmH_2_O	103.4 (38.4)	101.1 (34.6)	81.8 (39.7)	108.0 (40.7)	116.8 (40.2)	<0.001 (R vs. O <0.001; RE vs. O = 0.006; CHD vs. R < 0.001; & RE vs R < 0.001)
MEP, % pred	57.5 (18.2)	56.76 (17.3)	49.4 (20.2)	59.3 (18.3)	61.6 (18.7)	0.006 (R vs. O = 0.04; CHD vs R = 0.006; RE vs. R < 0.001)
Stability index	4.3 (2.3)	4.3 (2.8)	4.3 (3.6)	4.8 (2.8)	3.5 (1.6)	0.008 (RE vs. CHD) = 0.006)
Daily steps, n/day	5313.8 (3600.1)	5203.0 (3626.4)	4558.7 (2709.1)	5452.6 (3823.5)	5888.9 (3520.5)	0.185

Note: 6MWD: 6 min walk distance; BMI: Body mass index; CABG: Coronary artery bypass graft; CAD: Coronary artery disease; CHD: Chronic heart diseases; CHF: Chronic heart failure; COPD: Chronic obstructive pulmonary disease; CRF: Chronic respiratory failure; FEV1: Forced expiratory volume at 1st second; FVC: Forced vital capacity; ILD: Interstitial lung disease; LVEF: Left ventricle ejection fraction; MEP: Maximal expiratory pressure; MIP: Maximal inspiratory pressure; MVC: Maximal voluntary capacity; Obstructive: Chronic airway diseases; Other RS: Other restrictive syndromes; OHS: Obesity-hypoventilation syndrome; PTCA: Percutaneous transluminal coronary angioplasty; Restrictive: Pulmonary restrictive diseases; REVASC: Recent myocardial revascularization.

**Table 2:** Percentage of patients with impairment in baseline assessments.

	All	Obstructive	Restrictive	CHD	REVASC	*p*-Value
6MWD < 60% of pred.	33.2	36.6	52.7	29.9	15.4	<0.001 (RE vs. O <0.001; CHD vs. R = 0.010; RE vs. R <0.001)
MVC quadriceps < 60% of pred.	22.2	23.7	29.1	20.8	15.4	0.210
MVC biceps < 60% of pred.	6.77	6.25	12.73	5.84	6.59	0.330
MIP < 60% of pred.	21.4	19.1	32.7	20.1	25.3	0.174
MEP < 60% of pred.	57.3	58.1	65.4	55.8	51.6	0.410
Risk of fall index	36.9	35.3	36.4	41.6	35.16	0.592
Reduced physical activity	29.0	31.9	30.9	27.3	20.9	0.210

Note: 6MWD: 6 min walk distance; CHD: Chronic heart diseases; MEP: Maximal expiratory pressure; MIP: Maximal inspiratory pressure; Obstructive: Chronic airway diseases; Restrictive: Pulmonary restrictive diseases; REVASC: Recent myocardial revascularization. For 6MWD, lower and upper limb muscles, MIP, MEP, and balance, a severe impairment was defined when the measured value was lower than the 60% of the expected value. Reduced physical activity was defined as less than 3300 steps/day.

**Table 3:** Rate of prescription of program components by diagnosis group.

	All	Obstructive	Restrictive	CHD	REVASC	*p*-Value
Constant-load cycling	61.3	54.1	45.4	70.1	75.8	<0.001
Interval training cycling	33.5	40.3	47.3	27.9	18.7	<0.001
Treadmill training	4.4	5.0	5.4	1.9	5.5	0.410
Arms endurance training	0.7	1.2	1.8	0	0	0.320
General strengthening	82.1	81.9	74.5	83.1	89.0	0.160
Selective leg strengthening	11.3	13.1	14.5	12.3	5.5	0.220
Selective arm strenghtening	4.0	3.1	5.4	1.9	5.5	0.390
Selective arm+leg strengthening	2.5	1.9	5.4	2.6	0	0.160
Balance training	29.1	31.3	30.6	37.8	5.7	<0.001
Inspiratory muscle training	20.8	18.4	27.3	19.6	26.9	0.197
Pedometer	27.7	29.7	30.6	29.4	15.9	0.067

Note: CHD: Chronic heart diseases; Obstructive: Chronic airway diseases; Restrictive: Pulmonary restrictive diseases; REVASC: Recent myocardial revascularization.

**Table 4:** Post training delta changes in outcomes measures (delta absolute value and delta % of baseline).

	All	Obstructive	Restrictive	CHD	REVASC	*p*-Value
**6MWD, meters** **% of baseline**	29.3 (59.3) 8.13	23.9 (60.0) * 7.03	29.9 (53.0) * 9.16	28.6 (59.6) * 8.12	48.7 (56.1) * 11.43	0.012 (RE vs. O = 0.006)
**Quadriceps MVC, Kg** **% of baseline**	3.0 (8.0) * 14.0	2.9 (8.0) 13.6	4.0 (6.9) * 20.2	3.1 (7.5) * 13.4	2.8 (9.5) * 12.8	0.850
**Biceps MVC, kg** **% of baseline**	2.1 (4.5) * 12.0	1.9 (4.0) * 12.0	1.4 (4.9) 9.1	2.2 (5.1) * 11.1	3.1 (5.0) * 15.4	0.160
**MIP, cmH_2_O** **% of baseline**	5.8 (13.7) * 10.4	4.6 (12.5) * 7.9	0.9 (11.4)4.1	7.8 (15.0) * 15.6	9.6 (15.4) * 14.9	0.001 (RE vs. O = 0.030; CHD vs R = 0.020; RE vs R = 0.006)
**MEP, cmH_2_O** **% of baseline**	8.5 (27.6) * 11.8	8.5 (27.4) * 12.0	12.6 (30.9) * 19.0	4.9 (26.0) * 7.2	12.3 (28.6) * 14.2	0.220
**Risk of fall index** **% of baseline**	−0.6 (1.5) * −4.3	−0.6 (1.6) * −3.6	−0.7 (1.3) * −4.1	−0.6 (1.5) * −6.1	−0.4 (1.2) 4.0	0.900
**Daily steps, n/day** **% of baseline**	−132.5 (3051.6) * −2.4	−447.2 (2618.6) * −8.6	−944.5 (1986.0) * −20.7	−77.2 (3701.1) −1.4	1087.2 (3297.1) * 18.4	0.004 (RE vs. O = 0.006; RE vs R = 0.040)

Note: 6MWD: 6 min walk distance; CHD: Chronic heart diseases; MEP: Maximal expiratory pressure; MIP: Maximal inspiratory pressure; MVC: Maximal voluntary capacity; Obstructive: Chronic airway diseases; Restrictive: Pulmonary restrictive diseases; REVASC: Recent myocardial revascularization. Data are expressed as mean (standard deviation). * *p*-value <0.05 pre to post variation within each group.

## Supplementary Materials

The following are available online at https://www.mdpi.com/1660-4601/17/3/1016/s1.
