# Supplementary material for: Therapist Driven Rehabilitation Protocol for Patients with Chronic Heart and Lung Diseases: A Real-Life Study
Source: Int J Environ Res Public Health. 2020 Feb 5;17(3):1016. doi: 10.3390/ijerph17031016 (PMC7037983; doi:10.3390/ijerph17031016)
Supplement: Supplementary file 1 [file ijerph-17-01016-s001.pdf]

Therapist Driven Personalized Rehabilitation Protocol for Patients with Chronic Heart and Lung Diseases: A Real-Life Study

Supplementary Materials

The organization of the Cardio-Pulmonary Physical therapy Service was modified in order to allow the administration of the new algorithm to all admitted patients. The safety of the algorithm was investigated through the evaluation of major and minor side-effects. Feasibility was investigated considering the number of cases in which the prescription derived according to the algorithm required modifications by the physiotherapist (e.g., modification of the type of training, its characteristics, inability to administer certain treatments, etc.); the time per patient (in minutes) required by the physiotherapists to administer all tests required by the algorithm and obtain the prescription of exercise was estimated as well. Type and number of exercise prescriptions administered (constant-load training; interval training; treadmill training; general strengthening; specific strengthening of upper and/or lower limbs; balance training; inspiratory and/or expiratory muscle strengthening; and walking with pedometer) were compared with those prescribed during the 24 months (from 1st August 2014 to 1st January 2015) preceding the introduction of the new algorithm.
The sensitivity of the driven protocol algorithm was assessed through the analysis of the pre-to-post variation of each measure included in the initial and final assessment.
The algorithm was developed stepwise, as described subsequently.

1) Guidelines selection and data collection

Italian and European available statements and guidelines on exercise-based cardiac and pulmonary rehabilitation were screened by two researchers independently (CS and MP), and the following data were collected on a database:

- A list of patients' main impairments and limitations in the field of exercise and physical activity (e.g, effort intolerance, breathlessness, reduction of peripheral muscle strength, etc);
- Assessment tools to quantify any specific limitation and outcome measures;
- Goals to be reached in order to overcome each limitation;
- Best-practice programs to reach a specific goal;
- Characteristics of each program (e.g. frequency, intensity, type, duration).

Data collected by the two reviewers were compared and disagreement between them was resolved by discussion and consensus. If impairments were related to common issues and could be overcome with similar programs, they were clustered into the same group (e.g. exercise-induced breathlessness, exercise-induced muscle fatigue, exercise anxiety and exercise-induced oxygen desaturation were all related to reduced exercise capacity and were clustered together).

2) Data synthesis

Thereafter, data were analyzed and synthesized: for each impairment (e.g. exercise intolerance), specific exercise-based goals were defined (e.g. to improve exercise tolerance) and assessment tools were chosen in order to quantify any specific limitation (e.g. six-minute walking test). Assessment tools were chosen only if published validation studies reporting the equations used to calculate the predicted value existed. Thereafter, specific training program pathways (in terms of modalities, volume and intensity) aiming to reach the specific goal and overcome the limitation were defined (e.g. continuous or interval cycling training). Then, a prescriptive algorithm was constructed by the expert panel. When incomplete or missing information could not be retrieved from the literature, the two senior physicians and the senior physiotherapist gave their expert opinion to complete missing items of the algorithm, considering the literature.
